# High- or Low-Yielding F_2_ Progeny of Wheat Is Result of Specific *TaCKX* Gene Coexpression Patterns in Association with Grain Yield in Paternal Parent

**DOI:** 10.3390/ijms25063553

**Published:** 2024-03-21

**Authors:** Karolina Szala, Marta Dmochowska-Boguta, Joanna Bocian, Wacław Orczyk, Anna Nadolska-Orczyk

**Affiliations:** Plant Breeding and Acclimatization Institute—National Research Institute, Radzikow, 05-870 Blonie, Polandm.dmochowska@ihar.edu.pl (M.D.-B.);

**Keywords:** paternal inheritance, epigenetic imprinting, expression pattern, *TaCKX*, *NAC2*, cytokinins, wheat yield, breeding

## Abstract

Members of the *TaCKX* gene family (GFM) encode oxidase/dehydrogenase cytokinin degrading enzymes (CKX), which play an important role in the homeostasis of phytohormones, affecting wheat development and productivity. Therefore, the objective of this investigation was to test how the expression patterns of the yield-related *TaCKX* genes and *TaNAC2-5A* (*NAC2*) measured in 7 days after pollination (DAP) spikes and the seedling roots of parents are inherited to apply this knowledge in the breeding process. The expression patterns of these genes were compared between parents and their F*_2_* progeny in crosses of one mother with different paterns of awnless cultivars and reciprocal crosses of awned and awnless lines. We showed that most of the genes tested in the 7 DAP spikes and seedling roots of the F_2_ progeny showed paternal expression patterns in crosses of awnless cultivars as well as reciprocal crosses of awned and awnless lines. Consequently, the values of grain yield in the F_2_ progeny were similar to the pater; however, the values of seedling root mass were similar to the mother or both parents. The correlation analysis of *TaCKX* GFMs and *NAC2* in spikes and spikes per seedling roots reveals that the genes correlate with each other specifically with the pater and the F_2_ progeny or the mother and the F_2_ progeny, which shape phenotypic traits. The numbers of spikes and semi-empty spikes are mainly correlated with the specific coexpression of the *TaCKX* and *NAC2* genes expressed in spikes or spikes per roots of the pater and F_2_ progeny. Variable regression analysis of grain yield and root mass with *TaCKX* GFMs and *NAC2* expressed in the tested tissues of five crosses revealed a significant dependency of these parameters on the mother and F_2_ and/or the pater and F_2_ progeny. We showed that the inheritance of yield-related traits depends on the specific cooperative expression of some *TaCKX* GFMs, in some crosses coupled with *NAC2*, and is strongly dependent on the genotypes used for the crosses. Indications for parental selection in the breeding of high-yielding lines are discussed.

## 1. Introduction

Cytokinins are an important group of phytohormones that regulate the basic processes of growth and plant development [1,2,3,4]. Their key role in plant productivity has already been documented in many research studies [5,6,7,8,9,10].

The cytokinin content in plant organs is regulated mainly by metabolic processes, as well as their transport [11,12,13]. The predominant role in metabolic processes is played by the irreversible degradation of cytokinins by CKX enzymes, which can regulate rice grain yield [14,15,16,17]. In wheat, CKX enzymes are encoded by the *TaCKX* GFM represented by 13 basic genes; however, 11 of them have their homoeologs in each of the three subgenomes of wheat, A, B, and D [18]. A significant decrease in expression by silencing the *HvCKX1* and *TaCKX1* genes using RNAi technology in barley and wheat showed that a low level of expression of these genes determines high yield [19,20]. Similarly, the silencing of the *TaCKX2.1*, *TaCKX2.2.1*, and *TaCKX2.2.2* genes regulates yield-related traits in different ways, which are dependent on awned or awnless wheat spikes [21,22]. The change in the expression level of one of the *TaCKX* genes resulted in a decrease or increase in the expression levels of other genes. Therefore, the silencing of one of the *TaCKX* genes resulted in a specific expression pattern of other *TaCKX* genes, which regulates the content of cytokinins and other phytohormones, as well as yield-related traits [20,21,22]. There is a wide range of natural variability between the expression levels of the *TaCKX* genes and their patterns of coexpression in different cultivars and breeding lines [23]. Since the *TaCKX* gene expression pattern is related to wheat yield parameters, it was interesting to check how this pattern is inherited. In our earlier research with *TaCKX* GFMs, we also included *TaNAC2-5A*, which encodes the NAC-type transcription factor [22,23,24,25]. The *TaNAC2-5A* was found to increase wheat yield by controlling the nitrate response [26,27], and in our research, the gene was coexpressed with selected *TaCKX* GFMs and was correlated with several yield-related traits.

The most common way of inheritance of expression patterns, observed in diploid species, referred to as ‘parental expression additivity’ is the average of expression of the parental genes typically observed in diploid species [28]. Exceptions from this scheme were mainly observed in the progeny of polyploid species, in which the expression level was similar to that of one of the parents or was lower or higher than in both parents, or unequal. Such nonadditive gene expression levels associated with phenotypic heterosis in F_1_ plants have already been reported and reviewed [29,30,31,32,33]. These deviations from the general role are the result of different factors, such as epigenetic regulation by transcription factors [34,35,36,37,38], the balance of gene dosage [39,40], small interfering RNAs (sRNA) [36,41], histone modifications [42], R-loop formation [43], or distally acting factors [31]. Furthermore, noncoding RNAs have been described as regulators of the development of shoots and grains in barley [44] and dominant epigenetic regulators of early meiotic stages in wheat, ensuring reproductive success [45]. Small noncoding RNAs were involved in photosynthesis, glycolysis, hormone biosynthesis, and cellular homeostasis; however, long noncoding RNAs increased the expression of nearby genes.

The first exception from the common way of inheritance of expression pattern is when the expression level is similar to that of one parent, as described by Yoo et al. [28]; expression level dominance is more commonly called genomic imprinting [42,46,47,48,49,50]. It takes place when genes adopt the parent-of-origin expression pattern. One of the functions of this phenomenon is the regulation of seed dormancy through epigenetic mechanisms in gametes [50]. Genes with triple repressive marks H3K27me3/H3K9me2/CHGm remained stably imprinted. Some genes in cereals showed conserved imprinting associated with positive selection pressure [46]. Such parent-of-origin gene expression could affect a single gene or a group of genes. The transcriptome-wide identification of allele-specific imprinting genes in the maize embryo and endosperm of three reciprocal crosses revealed their involvement in nutrient transport, signaling pathways, and the transcriptional regulation of kernel development [51].

Most imprinted genes were described as maternally expressed and inherited [52,53,54], suggesting their predominant role in early cereal grain development [55,56]. There is rare evidence for paternally expressed imprinted genes. In Capsella, paternally imprinted genes allow for the overcoming of hybridization barriers [57]. In maize, the imprinted *dosage-effect defective1* (*ded1*) locus has been identified as a paternal regulator of seed size [58]. *Ded1* encodes one of the MYB transcription factors and is expressed specifically during early endosperm development, resulting in the repression of late grain-filling genes.

Most of the described epigenetic changes are regulated during plant development and are called developmental epigenetics. However, some of these changes in DNA methylation are stably inherited between generations, which is called transgenerational epigenetics [59], and most of them occur during seed formation [34].

All reports cited above concentrated on the molecular aspects of developmental and transgenerational epigenetics, usually up to the generation of F_1_. Since crossbreeding and selection are the basic steps in breeding, and the expression pattern of *TaCKX* genes regulates yield-related traits, it was important to check how the expression patterns of these genes together with yield-related traits are inherited. In our primary report on the transgenerational inheritance of agronomically important genes, we tested the expression patterns of *TaCKX* genes and *TaNAC2-5A* in segregating the F_2_ generation of reciprocal crosses of polyploid wheat [24]. We documented that some of them were paternally imprinted, together with the yield parameter. The research was conducted based on reciprocal crosses of selected, awnless cultivars. In this article, we continue research on the inheritance of patterns of expression of *TaCKX* genes and *TaNAC2-5A* between parents and the F_2_ generation of crosses of one mother with different paterns of awnless cultivars and reciprocal crosses of awned and awnless lines. For the first time, variable regression analysis is used to find a significant dependence of *TaCKX* GFMs and *NAC2* expressed in tested tissues on grain yield and root mass in the mother and F_2_ and/or the pater and F_2_ progeny.

## 2. Results

### 2.1. Crosses of One Mother with Different Paterns

#### 2.1.1. Crosses of One Mother with Different Paterns Lead to Different Patterns of *TaCKX* Expression and Data of Yield-Related Traits in F_2_

**Figure 1:**
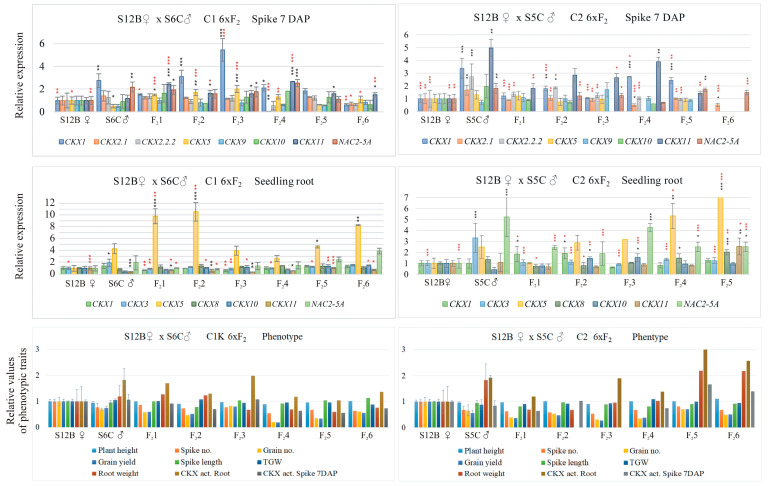
*TaCKX* GFM and *NAC2* expression patterns in 7 DAP spikes, seedling roots, and phenotypic traits in mother, pater, and their six F_2_ progeny, from crosses of S12B × S6C (C1*) and S12B × S5C (C2). Data represent mean values with standard deviation and are related to mother set as 1.00. Black and red asterisks indicate statistical significance compared to maternal parent or paternal parent, respectively (* 0.05 > *p* ≥ 0.01, ** 0.01 > *p* ≥ 0.001, *** *p* < 0.001). Data for C1 cross were already presented in Szala et al. [24], where S12B was component of reciprocal cross.

The relative values (related to mother = 1.0) of the expression profiles of the *TaCKX* family genes in 7 DAP spikes, seedling roots, and phenotypic characteristics of the mother S12B crossed with the pater S6C (C1) in the F_2_ progeny are different from the profiles of the same traits in crosses of the same mother S12B crossed with another pater, S5C (C2) (Figure 1; the measured values of phenotypic traits are in Table S1). Similarly, crosses of another mother, S6C, with three different paterns, S3C (C3), S12B (C4), and S5C (C5), lead to different patterns of *TaCKX* expression and yield-related trait profiles (Figure S1). These differences are mainly influenced by the pater. For example, in S12B crossed with S6C, the high relative expression of *TaCKX1* and *NAC2* in the 7 DAP spikes of the pater is also observed in the F_2_ progeny. Furthermore, the high expression of *TaCKX5* and *NAC2* and the low expression of *TaCKX11* in the seedling roots of the S6C pater are also inherited in F_2_. Similarly, in S12B crossed with S5C, the high relative expression of *TaCKX1* and *11* in spikes and *TaCKX5* and *NAC2* in pater seedling roots was observed in F_2_ progeny. In both crosses, the activity of CKX in the roots of the pater and F_2_ progeny was increased; however, the yield that included the grain number of the pater and F_2_ progeny was decreased compared to the activity of CKX and the mother’s yield.

The expression profiles of the *TaCKX* genes in 7 DAP spikes, seedling roots, and the phenotypic traits of S6C crossed with three paterns, S3C (C3), S12B (C4*), and S5C (C5), are presented in Figure S1. Similarly to the expression results above, the patterns of most *TaCKX* genes and *NAC2* in the spikes and roots of the F_2_ progeny are more comparable to the pater. Unfortunately, the grain yield in the F_2_ of each cross of the S6C as a mother with one of the three paterns was lower than in the mother and comparable to the pater, and this result was opposite to a much higher root mass.

#### 2.1.2. Crosses of One Mother with Different Paterns Show That the Expression Patterns of Most of the *TaCKX* GFM and the Yield Are Mainly Inherited from the Pater

The high or low expression of the *TaCKX* GFM in 7 DAP spikes and seedling roots and the yield-related traits in the mother, pater, and F_2_ progeny of C1 to C5 crosses are presented in Table 1. The data of the F_2_ progeny show a similar tendency to increase or decrease as colored paterns are in red, and those similar to those of the mother are colored green. Most of the gene expression levels in both the 7 DAP spikes and the seedling roots, as well as the yield and CKX activity data, are comparable to the pater (red). For example, in the C3 cross, S6C (mother) showed a low level of expression of *TaCKX5*, *11*, *9*, and *10* and a higher level of expression of *TaCKX2.1* and *NAC2* in 7 DAP spikes, just opposite to S3C, which is the paternal component of this cross. In their F_2_ generation, *TaCKX11* and *10* were highly expressed as in the pater. In the seedling roots of S6C, the expression of *TaCKX5* and the expression of *NAC2* were very low, and the expression of *TaCKX3* was high, opposite to the pater, S3C; however, in F_2_, the expression level of *TaCKX11* and *8* was very high and high, respectively, and the expression level of *TaCKX3* was low, as in the pater. Similarly, in other crosses, the expression patterns of the *TaCKX* GFM and *NAC2* in the spikes and roots of F_2_ were inherited from the pater. The F_2_ progeny in crosses of C1 and C2, where S12B was a better-yielding mother component, and C5, where S6C was the mother with a better yield, showed a decrease in yield comparable to the pater. The decrease in yield was in opposition to the increase in root mass in crosses of S6C as the mother with S3C, S12B, and S5C. However, in the first two crosses, the mass of the root in F_2_ was higher than in both parents, and in the third cross, it was comparable to the high mass of roots in the pater. On the contrary, only in one C1 cross, the expression of *TaCKX5* (together with *TaCKX9*) in the mother spike was higher than in the pater spike, and in the F_2_ progeny, it was similar to that of the mother. Interestingly, the high expression of *TaCKX5* in F_2_ was accompanied by the high expression of *TaCKX11* (and paternal *TaCKX1* and *NAC2*).

#### 2.1.3. How Expression Levels of *TaCKX* GFMs and *NAC2*, CKX Activity in 7 DAP Spikes and Roots, and Yield-Related Traits of the Mother and the Pater Were Correlated with These Traits in F_2_

The positive or negative correlations between *TaCKX* GFMs and *NAC2* expression in 7 DAP spikes and in seedling roots, CKX activity, and the yield-related traits of the mother and F_2_ or the pater and F_2_ in different crosses are illustrated in Table 2. The correlation coefficients are presented in Table S2.

The expression of *TaCKX1* was positively correlated with the expression of *TaCKX5* and *1* in the 7 DAP spikes of the mother and F_2_ (Table 2, blue) but not in the pater and F_2_ progeny. However, in the 7 DAP spikes of the pater and F_2_ progeny, *TaCKX1* was positively correlated with the expression of *TaCKX2.1* and *2.2.2* (Table 2, green). Furthermore, the levels of expression of these genes (*TaCKX2.1* and *2.2.2*) were positively correlated with *NAC2* only in P + F_2_. Another specific positive correlation of P + F_2_ was between *TaCKX2.2.2* and *10*, and a negative correlation was between *TaCKX2.1* and *9*. In contrast, in M + F_2_, *TaCKX2.2.2* was positively correlated with *9* or *11*, *TaCKX2.1* with *10*, and *TaCKX10* with *11* and *NAC2*. Others, such as *TaCKX2.2.2* alone, *TaCKX5* and *9* or *TaCKX5* and *10* or *TaCKX5* and *11*, *TaCKX10* and *11*, and *TaCKX2.1* with *NAC2*, are positively correlated in both M + F_2_ and P + F_2_.

The expression data of *TaCKX* GFMs and *NAC2* in spikes correlated with those tested in roots (*TaCKX1*, *3*, *5*, *8*, *10*, *11*, and *NAC2*) are also different in both groups, M + F_2_ and P + F_2_. Expressed in spikes, *TaCKX1* was positively correlated, depending on the cross, with *TaCKX1*, and with *TaCKX5* and *10* or negatively with *TaCKX9* or *TaCKX11* only in M + F_2_. Specific to P + F_2_, positive or negative correlations were between *TaCKX5* and *5*, *TaCKX5* and *1*, *TaCKX9* and *1*, *TaCKX2.1* and *11*, *NAC2* and *TaCKX3*, and *TaCKX11* and *NAC2*. The expression of *TaCKX9*, *10*, and *11* in spikes was correlated with the expression of multiple *TaCKX* and *NAC2* genes in roots. All three and some others are strongly correlated with *TaCKX8* and *11* in M + F_2_ or P + F_2_. For example, *TaCKX9* was negatively correlated with *TaCKX8* and *11* but positively correlated with *TaCKX10* in the roots of both groups.

Among yield-related traits, PH and SN were positively correlated with CKX activity in the spikes of P + F_2_ and M + F_2_ (Table S2), and PH data were correlated with the expression of *TaCKX1* and *11* or with *TaCKX11* alone in the spikes of M + F_2_. Furthermore, PH was also positively correlated with spike *TaCKX1* per root *TaCKX5* and *10* and with spike *TaCKX11* per root *TaCKX8* or negatively with spike *TaCKX11* per root *TaCKX1* and *3* of M + F_2_. The ES trait was correlated with the expression of *TACKX1* in M + F_2_ exclusively. In another cross (M2 + P_2_), ES together with SN was also correlated with *TaCKX2.1* and *NAC2* or SN with *TaCKX2.2.2* in both M + F_2_ and P + F_2_. The expressions in the spikes of M + F_2_, *TaCKX2.1*, and *TaCKX10* with *11* and *NAC2* were positively correlated with TGW. GY was negatively correlated with *TaCKX11* and *NAC2*, and *TaCKX5* and *9* in the M + F_2_ of two crosses. Both PH and ES, as well as RM, GN, and GY, were correlated with the expression of selected *TaCKX* and *NAC2* genes in group M + F_2_ exclusively and with TGW predominantly. SN and SES were observed more frequently but not exclusively in the group of P + F_2_ from different crosses. SN was positively correlated with *TaCKX2.1* in P + F_2_, and both SN and SES were strongly negatively correlated with spike *TaCKX9* per root *TaCKX8* and *11* in both M + F_2_ and P + F_2_ or strongly negatively correlated with spike *NAC2* per root *TaCKX3* in P + F_2_. RM was positively correlated with the expression of *TaCKX5*, *11* in spikes (M + F_2_ and P + F_2_), negatively correlated with spike *NAC2* per root *NAC2* in P + F_2_, and positively correlated with *NAC2* in M + F_2_.

The correlation coefficients between *TaCKX* and *NAC2* expression, CKX activity, and yield-related traits are related to both parents or the mother or pater separately.

### 2.2. Reciprocal Crosses of Awned × Awnless Lines (C6, C7)

#### 2.2.1. Reciprocal Crosses of Awned and Awnless Lines Lead to Opposite *TaCKX* GFM and *NAC2* Expression Patterns and Yield-Related Traits in F_2_

**Figure 2:**
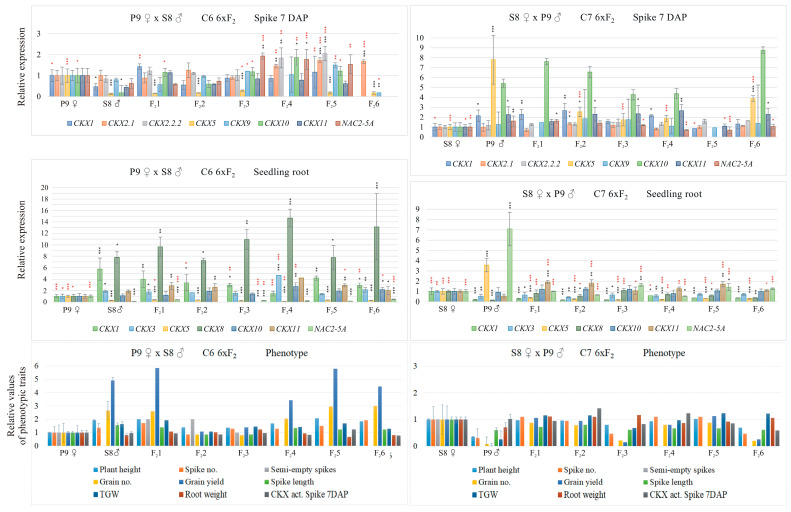
*TaCKX* GFM and *NAC2* expression patterns in 7 DAP spikes, seedling roots, and phenotypic traits in mother, pater, and their six F_2_ progeny, from crosses of awned mother and awnless pater, P9 × S8 (C6), and awnless mother and awned pater, S8 × P9 (C7). Data represent mean values with standard deviation and are related to mother set as 1.00. Black and red asterisks indicate statistical significance compared to maternal parent or paternal parent, respectively (* 0.05 > *p* ≥ 0.01, ** 0.01 > *p* ≥ 0.001, *** *p* < 0.001).

In the first cross, the awned spike line representing the mother was crossed with the awnless line (C6). In the reciprocal cross, the awnless line was the mother component, and the awned line was the pater component (C7). The expression of the *TaCKX* GFM and *NAC2* in 7 DAP spikes and seedling roots, as well as yield-related traits in the mother, pater, and their six F_2_ progeny in C6 and C7 crosses, is presented in Figure 2. The awned mother line showed higher expression of almost all tested *TaCKX* genes, as well as lower yield-related parameters compared to the pater. The gene with the lowest expression level in pater spikes, related to the mother (=1.00), was *TaCKX5* and then *TaCKX10* and *1*. *TaCKX2.1* was at the same level in both parents. The expression of *TaCKX5* in F_2_ remained at the same very low level as in the pater; for *TaCKX1*, the expression level was similar or slightly higher than in the pater, and in the case of *TaCKX10*, it was several times higher compared to the pater. In seedling roots, the awnless pater showed several times higher expression of *TaCKX1* and 8 and much lower expression of *TaCKX5* and *NAC2* than in the awned mother. This pattern of expression of *TaCKX1*, *8*, *3* and *TaCKX5* and *NAC2* (much higher or much lower expression levels than in the mother, respectively) was observed in the F_2_ progeny. Yield-related traits such as grain number and grain yield in four of the six F_2_ progeny were similar to the pater and much higher than in the mother.

The expression of the *TaCKX* GFM and *NAC2* in 7 DAP spikes and seedling roots, as well as yield-related traits, in the awnless mother crossed with the awned pater (C7) were opposite to those of the C6 cross. The pattern of higher expression of *TaCKX1*, *5*, *10*, *11* in the 7 DAP spikes of the pater was transmitted to the F_2_ progeny. The very low expression of *TaCKX1*, *3* in the pater roots was also low in the F_2_ progeny. However, the very high expression of *TaCKX5* and *NAC2* was much lower in F_2_, and in the case of *TaCKX5*, it was even lower than in the mother. Very low parameters of grain number, grain yield, and TGW in the pater were in the F_2_ progeny slightly higher than in the pater or similar to the mother.

#### 2.2.2. Reciprocal Crosses of Awned and Awnless Lines Showed That the Expression Patterns of Most *TaCKX* GFMs and Yield Are Mainly Inherited from Pater

*TaCKX* GFMs with high or low relative expression in 7 DAP spikes, seedling roots, and the parameters of yield-related traits in the mother, pater, and F_2_ progeny of reciprocal C6 and C7 crosses are presented in Table 3.

Very low expression of *TaCKX5* in the 7 DAP spikes of the awnless, high-yielding pater (C6) was transmitted to the F_2_ progeny (red). Similar expression levels between the pater and F_2_ progeny in seedling roots were shown by highly expressed *TaCKX1* and *8* and lowly expressed *TaCKX5* and *NAC2*. The yielding parameters were very high in both the pater and F_2_ progeny.

In the C7 cross, where the pater was an awned component, the very high expression of *TaCKX5* in the 7 DAP spikes of the pater was lower in the F_2_ progeny but still higher than in the mother. Furthermore, the very high level of expression of *TaCKX10* and the high level of *TaCKX1* and *11* in the pater were similar in the F_2_ progeny. The low expression levels of *TaCKX1* and *8* in the roots of the awned pater were similar in the F_2_ progeny; however, the low expression level of *TaCKX5* was similar in the F_2_ progeny to the mother. In this cross, the yield-related traits of the F_2_ progeny of the very high-yielding awnless mother crossed with the very low-yielding awned pater were higher compared to the pater or on a level similar to that of the mother.

#### 2.2.3. The Correlation between the Expression of *TaCKX* GFM and *NAC2*, as Well as the Yield-Related Traits, in Reciprocal Crosses of Awned and Awnless Parents and Their F_2_ Progeny Indicates the Predominant Role of the Awned Component

The correlation between the *TaCKX* GFM and *NAC2*, as well as yield-related traits, in the groups of M + F_2_ and P + F_2_ of reciprocal crosses of awned and awnless parents is presented in Table 4. The correlation coefficients are presented in Table S3.

The expression levels of *TaCKX* GFM and *NAC2* in the spikes of M + F_2_ correlate positively with the expression levels of each gene in the spikes of P + F_2_ of the C6 and C7 crosses (Table 4, first row, and Table S3, yellow). The largest differences in the correlations of expression are between *TaCKX* GFM and *NAC2* in the spikes and roots of M + F_2_ and P + F_2_. Most genes expressed in the spikes of awned M + F_2_ correlate with the *TaCKX* GFM and *NAC2* in roots, starting from the positive correlations of spike *TaCKX1* with root *TaCKX5* and *NAC2* through the negative correlations of spike *NAC2* with root *TaCKX1* and *8*. The only negative correlation of spike *TaCKX1* with root *TaCKX10* was observed in both M + F_2_ and P + F_2_ (bold). Similarly, correlations between the *TaCKX* GFM and *NAC2* in roots were observed mainly in awned M + F_2_; however, the correlation between *TaCKX1* and *5* in M + F_2_ is negative, but in P + F_2_, it is positive.

Numerous correlations between *TaCKX* GFM and *NAC2* expression were observed in the spikes and roots of M + F_2_ and P + F_2_ of the reciprocal C7 cross, where M was awnless, and P was awned (Table 4). Many of them, such as the positive correlations between *TaCKX1* in spikes and *TaCKX5* in roots and *TaCKX5*, *9*, *10*, *11*, and *NAC2* in spikes and *TaCKX5* in roots, represented both awnless M + F_2_ and awned P + F_2_. However, many correlations between *TaCKX2.1*, *5*, *9*, *10*, *11*, and *NAC2* in spikes and *TaCKX8* in roots were positive in M + F_2_ but negative in P + F_2_, and the correlation between *TaCKX2.2.2* and *TaCKX11* was negative in both groups. However, the correlations between other *TaCKX* genes in the spike and the root differed in these two groups and were more frequent in P + F_2_, where, similar to C6, P was the awned parent.

The correlations between yield-related traits and the expression of the *TaCKX* GFM and *NAC2* in spikes and roots in the two groups of the M + F_2_ and P + F_2_ of C6 and C7 crosses are presented in Table 5.

Numerous correlations between yield-related traits and *TaCKX* GFM and *NAC2* expression were observed in both spikes and roots in the awned M + F_2_ or awned P + F_2_ of the C6 or C7 cross, respectively. On the contrary, in the C6 group of awnless P + F_2_ and the C7 group of awnless M + F_2_, there were only a few correlations between yield-related traits and the *TaCKX* GFM and *NAC2* in spikes: negative between root weight and *TaCKX1*, *2.1*, *2.2.2* in C6 P + F_2_, which was the same in M + F_2_; negative between the semi-empty spike number and *TaCKX2.1*, *2.2.2*, *10*, and *NAC2* and positive between spike length and *TaCKX1*, *9*, *11*, and *NAC2* (C6) or positive between spike length and *TaCKX1*, *2.1*, *5*, *9*, *10*, *11*, and *NAC2* (C7). Furthermore, in the awnless P + F_2_ of the C6 group, the spike number was correlated with the *NAC2* expressed in roots, and in the awnless M + F_2_, spike length was correlated with the root *TaCKX5*, *8*.

### 2.3. Stepwise Regression Analysis of Grain Yield and Root Mass with TaCKX GFMs and NAC2 Expression

**Figure 3:**
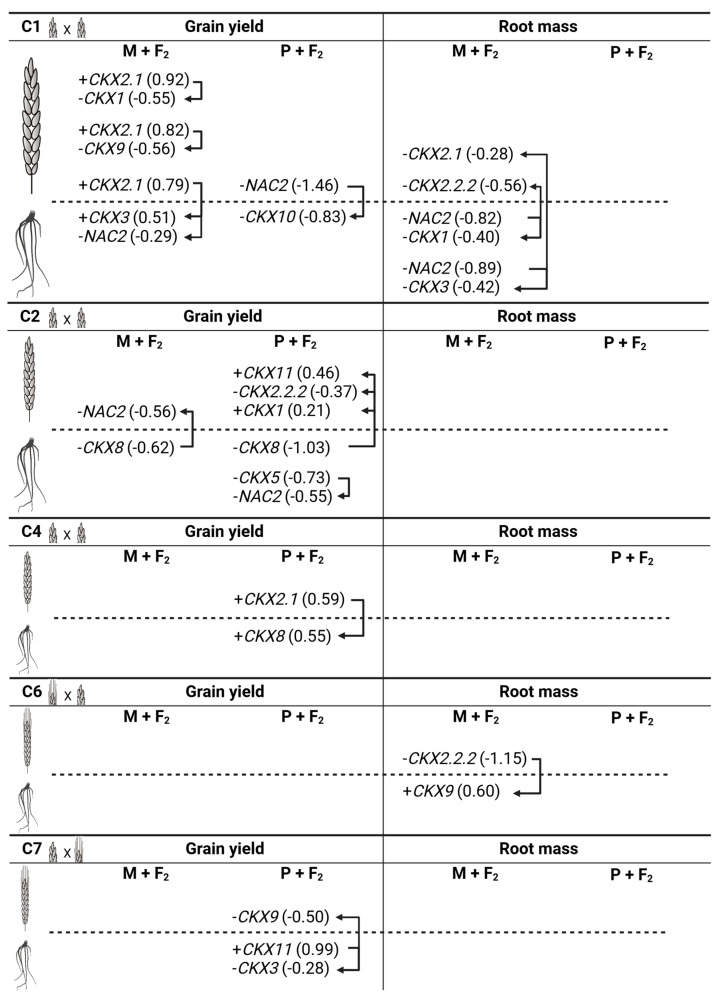
Map of dependent variable regression of grain yield and root mass with *TaCKX* GFMs and *NAC2* in mother and F_2_ and pater and F_2_ of different crosses.

The map of significant dependent variable regression of grain yield and root mass with *TaCKX* GFMs and *NAC2* in the mother and F_2_ and the pater and F_2_ in different crosses is presented in Figure 3.

The regression of grain yield and root mass was generally significant for a pair or three of genes in the mother and F_2_ and the pater and F_2_ in crosses of awnless cultivars (C1–C5). The most frequent for grain yield in 7 DAP spikes was the positive regression of *TaCKX2.1* (0.92) and negative *TaCKX1* (−0.55); the positive regression of *TaCKX2.1* (0.82) and negative *TaCKX9* (−0.56); the positive regression of *TaCKX2.1* (0.79) coupled with the positive regression of *TaCKX3* in roots (0.51) and negative with *NAC2* (−0.29). In the roots of the same cross, the negative regression of *NAC2* (−0.89) was coupled with the negative regression of *TaCKX3* (−0.42) and with the negative regression of *TaCKX2.1* (−0.28) in spikes. Furthermore, the negative regression of *NAC2* (−0.82) in the roots of M + F_2_ was coupled with the negative regression of root *TaCKX1* (−0.40) and spike *TaCKX2.2.2* (−0.56). However, the highest negative regression was for *NAC2* (−1.46) in the spikes of the pater and F_2_ coupled with root *TaCKX10* (−0.83). In the C2 cross, the negative regression of the spike *NAC2* was coupled with *TaCKX8* in the roots of the mother and F_2_; however, in the pater and F*_2_*, negative regression of the root *NAC2* (−0.55) was coupled with the negative regression of the root *TaCKX5* (−0.73). In the same group of the pater and F_2_ of the C2 cross, the negative high regression of the root *TaCKX8* (−1.03) was combined with the positive regression of the spike *TaCKX11* (0.46) and *TaCKX1* (0.21) and the negative regression of the spike *TaCKX2.2.2* (−0.37). In the C4 cross, the positive regression of the spike *TaCKX2.1* was coupled with the positive regression of the root *TaCKX8* but only in the pater and F_2_. The only regression coefficients for the grain yield of the C3 and C5 crosses were for the root *TaCKX5* (−0.23) coupled with the spike *TaCKX2.2.2* (−0.15) and for the root *TaCKX10* (+0.34) coupled with the spike *TaCKX10* (−0.09) in the mother and pater and F_2_.

The regression coefficients of grain yield with *TaCKX* GFMs and *NAC2* in the reciprocal crosses of awned and awnless cultivars were significant only in the pater and F_2_ when the pater was an awned component (C7). In this cross, grain yield showed positive regression with root *TaCKX11* (0.99) coupled with the negative regression of root *TaCKX3* (−0.28) and spike *TaCKX9* (−0.50). The mass of the root showed strong, negative regression coefficients with spike *TaCKX2.2.2* (−1.15) positively coupled with root *TaCKX9* (0.60) in the awned mother and F_2_ of the C6 cross.

## 3. Discussion

### 3.1. The Expression Patterns of the TaCKX Genes, TaNAC2-5A, and Grain Yield Are Inherited from the Paternal Parent and Are Genotype-Dependent

In our previous research [24], it was documented for the first time that the expression levels of most of the yield-related *TaCKX* genes, *TaNAC2-5A*, and grain yield were inherited in F_2_ from the paternal parent. The experiment was carried out using reciprocal crosses of awnless cultivars. This unexpected way of inheritance was proved in the present research based on other crosses: crosses of one mother with different parents of awnless cultivars and reciprocal crosses of awned and awnless lines. Interestingly, the yield values in F_2_ were similar to those of the pater as well. Therefore, presented by us in our previous research, the paternal pattern of inheritance of yield-related *TaCKX* gene expression and the yield in F_2_ generation was the first example of transgenerational paternal inheritance of these traits, and this research is a valuable confirmation of these data, including awned and awnless genotypes.

These different crosses of one mother with different paterns or reciprocal crosses of awned and awnless lines led to different patterns of the coexpression of *TaCKX* family genes, which together regulate yield-related traits in F_2_. The genotype dependency of specific, cooperative expression on yield-related traits was also very distinct in the silencing experiments of *TaCKX1* and *TaCKX2* genes in two cultivars, awnless Kontesa and awned Ostka [20,21,22]. The decreased silencing expression of one *TaCKX* gene mediates the decreased or increased expression of other *TaCKX* genes, regulating phytohormone content and yield-related traits in different ways. For example, in *TaCKX1* lines of the awnless wheat cultivar silenced by RNAi, the expression of *TaCKX11* was significantly decreased, but the expression of *TaCKX2* genes was significantly increased, resulting in a change in the content of cytokinins and other phytohormones and the obtaining of the wheat phenotype with a significantly increased spike and grain number but a decrease in TGW [20]. Interestingly, a similar pattern of expression of *TaCKX* genes in the silenced *TaCKX1* lines of awned cultivars regulated the content of cytokinins and other phytohormones differently, resulting in a significant increase in TGW and seedling root mass [22]. Similarly, the expression patterns in different crosses are regulated by the cooperation of the *TaCKX* genes in the same positive way and, for some, in the opposite way, and the obtained phenotype is a result of this cooperation. However, in all crosses of this research and a previous one [24], high-yielding F_2_ progeny was obtained, when a low-yielding mother was crossed with a high-yielding pater, suggesting that groups of paternally inherited *TaCKX* genes determinate, similar to the pater, high or low yield in F_2_.

### 3.2. Are Transcription Factors the Main Epigenetic Regulators in Wheat?

As reviewed in the introduction, any deviations from the non-Mendelian inheritance of a parent-of-origin expression pattern might be the effect of epigenetic regulation. The main factors of this epigenetic regulation are the balance of gene dosage [28], small interfering RNAs [36,41], noncoding RNAs [44,45], or *cis*- and/or *trans*-regulatory elements [34,35,36,37,38]. All of them might be involved in the regulation of these epigenetic types of expression patterns, especially in polyploid species. Noncoding RNAs have been described as the dominant epigenetic regulators of early meiotic stages in wheat, involved in photosynthesis, glycolysis, hormone biosynthesis, and cellular homeostasis [45], and in barley, they were found to regulate the development of shoots and grains [44]. Wheat *TaNAC* transcription factors are involved in the *cis*-regulation of selected *TaCKX* family genes [25]. Interestingly, one *TaNAC* can be involved in the regulation of the transcription of two to three *TaCKX* genes. For example, *TaNAC J-1* and *TaNAC94* are expected to regulate *TaCKX1*, *2.1*, and *5*; *NAC13a* was found to regulate *TaCKX2.2.1* and *10*; *TaNAC Br-1* was identified as the regulator of *TaCKX2.2.1*, *9* and *11*; and *TaNAC6D* binds to the *cis*-regulatory region of *TaCKX10*. We need to conduct more research to find which of these or other factors can be dominantly involved in the paternal inheritance of the expression pattern of *TaCKX* GFMs combined with yield.

### 3.3. TaCKX5 Plays a Major Role in Coexpression with Other TaCKX GFMs and TaNAC2-5A

As presented in this research, grain yield was inherited in F_2_ progeny as in the pater, regardless of awned or awnless spike cultivars. The same was documented in our previous research with awnless wheat cultivars [24]. It is difficult to indicate the common pattern of coexpression of *TaCKX* genes, which should be represented in the high-yielding pater to transmit this pattern of expression together with the yield to F_2_ generation. In the C6 cross of the awned × awnless line, the strong down-regulation of *TaCKX5* and *10* and the down-regulation of *TaCKX1* and *11* in the 7 DAP spikes of a high-yielding pater resulted in high-yielding F_2_. The coexpression of these genes is in agreement with silencing experiments in awned and awnless cultivars [21,22]. The down-regulation of *TaCKX1* expression was coordinated with the down-regulation of *TaCKX11* expression in both cultivars, as well as the decreased expression of *TaCKX5* in the awned, which resulted in an increased TGW, root mass, and grain yield in the awned [22]. However, in the case of the high level of coexpression of *TaCKX5* with *TaCKX9* and the decreased coexpression of *TaCKX1*, *2.1*, and *NAC2* of the high-yielding pater with the low-yielding mother in C4, the yield of the progeny in F_2_ was between both parents, and the mass of seedling roots was similar to the mother. These results demonstrate the importance of *TaCKX5* coexpression with others. This is reasonable since we found the highest expression of the *TaCKX5* gene among other *TaCKX* GFMs in inflorescences and seedling roots and very high in 0 DAP spikes and leaves [60]. As mentioned above, this gene might also be regulated by at least two TaNAC transcription factors [25].

### 3.4. Simultaneous Paternal Inheritance of the Expression Pattern of the TaCKX Genes and TaNAC2-5A with Grain Yield Is Not the Rule for Seedling Root Mass and Other Yield-Related Traits

Similarly to 7 DAP spikes, the expression pattern of the *TaCKX* genes and *NAC2* was inherited in the seedling roots of F_2_ as in the pater. However, unlike paternally inherited grain yield, seedling root mass was inherited in the F_2_ progeny similar to both parents or with values ranging between the two parents, or in two crosses (C3, C4) from the same S6C mother. In both crosses, the same pattern of high expression of *TaCKX8*, *10*, and *11* and decreased *TaCKX3* appeared in the roots of the pater and in the F_2_ progeny. Therefore, paternally inherited expression patterns in spikes and seedling roots influence grain yield; however, the mass of seedling roots is inherited from the mother or both parents. All these genes (*TaCKX3*, *8*, *10*, *11*), highly expressed in seedling roots, are also highly expressed in 0 DAP spikes and *TaCKX11* in inflorescences [60]; therefore, their coexpression in seedling roots influences grain yield.

The inheritance of coexpression patterns of *TaCKX* GFMs and *NAC2*, grain yield, and the mass of seedling roots could be explained based on the correlation coefficients of their expression in the pater and F_2_ compared to the mother and F_2_. *TaCKX1*, *2.1*, and *2.2.2* and *NAC2*, as well as *TaCKX 2.2.2* with *10*, and *CKX2.1* with *9* correlated with each other in 7 DAP spikes only in the pater and F_2_ progeny (not in the mother and F_2_ progeny) in tested crosses. However, some of them, like *TaCKX1* and *5*, *TaCKX10*, *11*, and *NAC2*, *TaCKX2.1* and *10*, and *TaCKX2.2.2* and *9* or *11* correlated with each other only in the mother and F_2_. Similar specific correlations of *TaCKX* GFMs and *NAC2* expressed in spike per these expressed in roots were documented for both groups. There are also a few examples of the same genes or gene pairs correlating with each other in both groups, the mother and F_2_ and the pater and F_2_. Generally, most yield-related traits, such as grain number, spike length, plant height, grain yield, and root mass and the most frequent TGW, were correlated with *TaCKX* GFMs and *NAC2* in spikes or spike per root in the mother and F_2_ group; however, some very important traits for total grain yield like spike number and semi-empty spike number were mainly correlated with selected spike or spike per root *TaCKX* GFMs and *NAC2* in the pater and F_2_ population. Therefore, depending on the crossed genotypes, the correlations between yield-related traits in the mother and F_2_ and the pater and F_2_ depended on the specific expression or more frequently the coexpression of several *TaCKX* and *NAC2* genes in 7 DAP spikes, as well as the seedling roots of the mother and F_2_ and the pater and F_2_.

### 3.5. Regression Analysis Proved a Significant Dependence of the Specific Expression Pattern on the Parameters of Yield-Related Traits in Mother and F_2_ or in Pater and F_2_

The map of dependent variable regression analysis of grain yield or root mass and the *TaCKX* GFM and *NAC2* expressed in the spikes and seedling roots of different crosses revealed a significant dependency of these parameters in the mother and F_2_ or in the pater and F_2_. Both traits were strongly dependent positively or negatively on groups of two to four tested genes expressed in spikes or seedling roots, specifically to both groups (mother and F_2_ or pater and F_2_). For example, grain yield in C1 is highly positively dependent on the spike *TaCKX2.1* and negatively on the spike *TaCKX1*, or the spike *TaCKX9*, or positively on the root *TaCKX3*, or negatively on the root *NAC2*, specific to the mother and F_2_ progeny; however, in the pater and F_2_ of the same cross, this trait was highly negatively dependent on the spike’s *NAC2* and the root’s *TaCKX10*. These parameters of the dependent variable regression were specific to the mother and F_2_ and/or the pater and F_2_ of each cross. Among the most frequent are the positive regression of grain yield and the spike *TaCKX2.1* and the negative regression of the grain yield and the spike or root *NAC2*. *TaCKX2.1* was isolated and characterized as a gene related to the grain number per spike by Zhang et al. [61]. In recombinant inbred lines, *TaCKX6a* [62], then renamed *TaCKX2.1-3D* [18], showed significant correlations with grain size, weight, and grain filling rate. However, in our investigation, the importance of positive or negative coregulation of *TaCKX2.1* with other *TaCKX* GFMs on yield-related traits was highlighted. In the silencing experiment, the down-regulation of wheat *TaCKX1* resulted in a strong down-regulation of *TaCKX1* and *11* and up-regulation of *TaCKX2.1* and others in both awnless and awned cultivars; however, it affected different yield-related traits, and only in the awned one, it resulted in a high-yielding phenotype [20,22]. Similarly, the spike *TaCKX2.1*, which is positively correlated with grain number, grain yield, spike number, spike length, and root mass, was coupled with other *TaCKX* GFMs, and in the case of grain yield, it was negatively correlated with the spike and root *TaCKX1* in the high-yielding F_2_ progeny [24]. In addition to this specific coregulation, *TaCKX1*, *2.1*, and *5* could be regulated by JUNGBRUNNEN 1-like TF, renamed TaNACJ-1 and TaNAC94 TF [25]. Based on gene ontology analysis, the *TaNACJ-1* takes part in the negative regulation of leaf senescence. This is also in agreement with the silencing experiments of the *TaCKX2* genes, which significantly increased chlorophyll content in the flag leaves of awned and awnless cultivars [21,22]. The second one, *TaNAC94*, can be involved in response to auxins, the positive regulation of asymmetric cell division, somatic stem cell division, root cap development, etc. [25]—traits that influence grain yield. And again, the silencing of *TaCKX2* genes in both awned and awnless cultivars affected not only the contents of cytokinins but also auxins, however, in different ways. The IAA content along with the active cytokinin content in the awnless cultivar was increased [21], but in the awned cultivar, the pattern of cytokinin content was different from the awnless one, and the IAA content was decreased [22]. Therefore, selected *TaNACs* could regulate the transcription of *TaCKX2* genes that influence phytohormone content and yield-related traits.

Another, the most dependent on grain yield in two crosses of one mother with two paterns, is *TaNAC2-5A*. This gene is specifically or not specifically correlated with others expressed in the spikes or spikes per roots *TaCKX* GFMs of all crosses and showed negative regression with grain yield and root mass, coupling positively or negatively with other *TaCKX* GFMs. The *TaNAC2-5A* belongs to the large family of *NAC* genes, which encode NAC-type transcription factors that are involved in the regulation of important agronomic traits [26,38,63,64]. The overexpression of the *TaNAC2-5A* enhanced root growth and increased the ability of the root to acquire nitrogen and, under field conditions, increased nitrate uptake and grain yield [26]. However, in a controlled environment, this gene is positively correlated with the activity of the CKX enzyme in seedling roots and negatively with tiller number [23], was expressed in roots together with *TaCKX3* and *8*, and was negatively correlated with root mass [24]. Here, the spike *TaNAC2-5A* together with the spike *TaCKX11* in the mother and F_2_ was negatively correlated with root mass, spike number, and grain yield, however, positively with TGW; but in the case of a negative correlation of spike *TaNAC2-5A* with root *TaCKX3* or root *TaNAC2-5A* in the pater and F_2_, negative correlations with the spike number and semi-empty spikes were observed. In summary, the expression of *TaNAC2-5A* in spikes and/or roots is coregulated by other genes from *TaCKX GFMs*. This coregulation is not direct, since we did not find TaNAC2-5A TF binding sites in the *cis*-regulatory sequences of *TaCKX* GFMs; however, the TF binding sites of the other five *TaNACs* were identified [25]. As reported by Li et al. [27], the NAC TF of *TaNAC2-5A* binds directly to the promoter of the nitrate transporter gene, *TaNRT2.5-3B*, playing a key role in seed vigor. Another *NAC2*, *OsNAC2* in rice, which can regulate the expression of auxin- and cytokinin-responsive genes, was shown to be an integrator of auxin and cytokinin pathways, playing a role in modulating root development [65], and through the ABA pathway delayed the germination of seeds [66].

## 4. Materials and Methods

### 4.1. Plant Material

Six common wheat breeding lines and cultivars (*Triticum aestivum* L.) of thirty-four breeding lines and cultivars previously studied [23] were selected for research as parents. The seeds were delivered by two plant breeding companies: Strzelce Ltd., Co.—IHAR-PIB Group (Strzelce, Poland) and Danko Hodowla Roslin Ltd. (Choryń, Poland). Parents, named by breeders S12B, S6C, S5C, S3C, P9, and S8, differ in expression levels of *TaCKX* GFMs and *NAC2* in seedling roots and 7 DAP spikes, and values of yield-related traits. They were used in five crosses (1) S12B × S5C (C2), (2) S6C × S3C (C3), (3) S6C × S5C (C5), (4) P9 × S8 (C6), and (5) S8 × P9 (C7) to obtain the F_1_ and F_2_ progeny. Each cross was represented by three plants from each parent and six individual, randomly selected F_2_ plants. Data from two crosses, S12B × S6C (C1) and S6C × S12B (C4), have already been published [24] but are shown here to compare with the new one.

### 4.2. Growing Conditions and Crossbreeding

The parent plants and the F_2_ plants were grown in the same growth chamber at the same time, to provide the same, controlled environment. Temperatures were maintained at 20 °C during the day and 18 °C at night, with a day/night cycle of 16 h of light followed by 8 h of darkness. The intensity of light was 350 μmol·s^−1^·m^2^. The plants were watered three times a week and fertilized once a week with Florovit, following the manufacturer’s guidelines.

The experimental tissue samples were collected from parental lines (3 plants per parent) and their six F_2_ progeny using the same methods as described in Szala et al. [24]. There were roots from 5-day-old seedlings, cut 0.5 cm from the base before replanting, and spikes from the same plants 7 days after pollination. All samples were taken at 9:00 a.m. and kept in freezer in liquid nitrogen at −80 °C until needed.

The crossbreeding was performed like in Szala et al. [24].

### 4.3. RNA Extraction, cDNA Synthesis, and RT-qPCR

Total RNA was extracted from the collected samples using TRI reagent according to the manufacturer’s instructions. RNA concentration and quality were determined according to Szala et al. [24]. High-quality RNA was used for cDNA synthesis using the RevertAid First Strand cDNA Synthesis Kit (Thermo Fisher Scientific, Vilnius, Lithuania). RT-qPCR assays were carried out for 10 genes, *TaCKX1*, *TaCKX2.1*, *TaCKX2.2.2*, *TaCKX3*, *TaCKX5*, *TaCKX8*, *TaCKX9*, *TaCKX10*, *TaCKX11*, and *TaNAC2-5A*, and all reactions were carried out in triplicate on a Rotor Gene Q thermal cycler (QIAGEN, Hilden, Germany) using HOT FIREPol EvaGreen qPCR Mix Plus (Solis BioDyne, Tartu, Estonia). Reaction conditions and pairs of primers for the genes studied were the same as in the previous publication and in Table S3. The expression of *TaCKX* genes was calculated using ADP-ribosylation factor as a normalizer.

### 4.4. Analysis of CKX Activity

CKX enzyme activity was measured on the same samples used for analysis of *TaCKX* gene expression according to the procedure developed by Frebort et al. [67] and was optimized for wheat tissues according to Szala et al. [24]. The procedure involved the extraction of plant material, incubation in a reaction mixture, and measurement of the concentration of the product. Total protein concentration was approximated by referring to the standard graph created using bovine serum albumin (BSA), following the Bradford method, as outlined by Bradford and Williams [68].

### 4.5. Measurement of Yield-Related Traits

The following yield-related traits were measured: the height of the plant, number of spikes, number of partially empty spikes, number of tillers, length of the spike, yield of grains, number of grains, weight of 1000 grains (TGW), and weight of 5-day-old seedling roots.

### 4.6. Statistical Analysis

For statistical analysis, Statistica version 13 software (TIBCO Software Inc., Palo Alto, Santa Clara, CA, USA) was utilized. Changes in significance were assessed through ANOVA variance analysis followed by the least significant difference (LSD) post hoc test. The correlation coefficients were calculated using parametric correlation matrices (Pearson test) or nonparametric correlation analysis (Spearman test). Progressive stepwise regression was calculated.

## 5. Conclusions

Expression patterns in spikes and seedling roots, as well as grain yield, in the F_2_ progeny are inherited like in the pater, while the mass of seedling roots is inherited from the mother or both parents. However, particular yield-related traits are regulated by specific, cooperative expression of a few *TaCKX* GFMs and in some crosses with *NAC2*. Both spike number and semi-empty spike number are mainly correlated with the specific coexpression of *TaCKX* and *NAC2* genes expressed in spikes or spikes per roots of the pater and F_2_ progeny, suggesting that these traits from the parent site are the main factors influencing grain yield. Regression analysis showed a strong dependence of grain yield or root mass on the coexpression of *TaCKX* genes and *NAC2* in the mother or pater, depending on the cross. Therefore, this specific cooperative expression is also very strongly dependent on the genotype. Parents and the F_2_ progeny of each cross used to have their own expression and gene cooperation pattern that influenced the traits in F_2_. Interestingly, in reciprocal crosses of awned and awnless lines and their F_2_ progeny, the predominant role was played by the awned component, regardless of whether it was the mother or the pater. Also, in these crosses, the grain yield was inherited after the pater. All of these data indicate that the pater component, which is selected for breeding, should be characterized by a specific *TaCKX* expression pattern and higher yield compared to the mother component.

## Figures and Tables

**Table 1:** *TaCKX* GFMs and *NAC2* expression levels in spikes and roots, and yield-related traits of mother (M), pater (P), and their F_2_ siblings from crosses of S12B × S6C, S12B × S5C, S6C × S3C, S6C × S12B, and S6C × S5C. Colors of characters indicate similar expression patterns and yield-related traits in F_2_ and pater (red) or in F_2_ and mother (green).

	**C1 = S12B × S6C = M1 × P1**		
	**M**	**P**	**F_2_**
*CKX* expression 7 DAP	*CKX1* ↓	*CKX1 * ↑↑	*CKX1*, *11*↑↑
	*NAC2* ↓	*NAC2 * ↑	*CKX5*, *NAC2 *↑
	*CKX5*, *9* ↑	*CKX5*, *9* ↓	
*CKX* expression root	*CKX5*, *NAC2* ↓↓	*CKX5*, *NAC2 *↑↑	*CKX5 *↑↑, *NAC2 *↑
	*CKX3* ↓	*CKX3*↑	
	*CKX11*, *10* ↑	*CKX11*, *10* ↓	*CKX11 * ↓
yield-related traits	yield ↑	yield ↓	Yield ↓
	CKX act. spike =	CKX act. spike =	CKX act. spike ↓↓
	root =↓	root = ↑	root =↓
	CKX act. root ↓↓	CKX act. root ↑↑	CKX act. root ↑↑
	**C2 = S12B × S5C = M1 × P2**		
	**M**	**P**	**F_2_**
*CKX* expression 7 DAP	*CKX1*, *2.2.2*, *11* ↓↓	*CKX1*, *2.2.2*, *11 *↑↑	*CKX1*, *11 *↑
	*CKX2.1*, *10*, *NAC2* ↓	*CKX2.1*, *10*, *NAC2* ↑	
*CKX* expression root	*CKX3*, *5*, *NAC2* ↓↓	*CKX3*, *5*, *NAC2 *↑↑	*CKX5*, *NAC2 *↑↑
	*CKX10* ↑	*CKX10* ↓	
yield-related traits	yield ↑	yield ↓	yield ↓↓
	CKX act. spike =	CKX act. spike =	CKX act. spike =
	root ↓↓	root ↑↑	root =↓↑
	CKX act. root ↓↓	CKX act. root ↑↑	CKX act. root ↑↑
	**C3 = S6C × S3C = M2 × P3**		
	**M**	**P**	**F_2_**
*CKX* expression 7 DAP	*CKX5*, *11* ↓↓	*CKX5*, *11 *↑↑	*CKX11 * ↑↑
	*CKX9*, *10* ↓	*CKX9*, *10 *↑	*CKX10 * ↑
	*CK2.1*, *NAC2* ↑	*CKX2.1*, *NAC2 *↓	*CKX2.1*, *NAC2* ↓
*CKX* expression root	*CKX5*, *NAC2* ↓↓	*CKX10*,*11 *↑↑	*CKX10*, *11 *↑↑
	*CKX3*↑	*CKX3* ↓	*CKX3* ↓
	*CKX8*, *10* ↓	*CKX8 * ↑	*CKX8 * ↑
yield-related traits	yield =	yield =	yield ↓
	CKX act. spike ↑↑	CKX act. spike ↓↓	CKX act. spike ↓
	root ↑	root ↓	root ↑↑
	CKX act. root =	CKX act. root =	CKX act. root ↓
	**C4 = S6C × S12B = M2 × P4**		
	**M**	**P**	**F_2_**
*CKX* expression 7 DAP	*CKX5*, *9* ↓	*CKX5*,*9*↑	*CKX5*, *9*, *10*,*11* ↑
	*CKX1*, *2.1*, *NAC2* ↑	*CKX1*, *2.1*, *NAC2* ↓	*CKX1*, *NAC2* ↓
*CKX* expression root	*CKX10*, *11* ↓↓	*CKX10*, *11 *↑↑, *CKX8 *↑	*CKX8*, *10*, *11 *↑↑
	*CKX3*, 5, *NAC2* ↑	*CKX3*, *5*, *NAC2* ↓	*CKX1*, * 3*, *NAC2* ↓
yield-related traits	yield ↓	yield ↑	yield =↓↑
	CKX act. spike =	CKX act. spike =	CKX act. spike =↓↑
	root↑↑	root ↓↓	root ↑↑
	CKX act. root ↑	CKX act. root ↓	CKX act. root =
	**C5 = S6C × S5C = M2 × P2**		
	**M**	**P**	**F_2_**
*CKX* expression 7 DAP	*CKX11* ↓↓	*CKX11 * ↑↑	*CKX11 * ↑↑
	*CKX2.2.2*, *5*, *10* ↓	*CKX2.2.2*, *5*, *10 *↑	*CKX2.2.2*,*5* ↑
	*NAC2* =	*NAC2* =	*NAC2* ↓
*CKX* expression root	*CKX11* ↓↓	*CKX11 * ↑↑	*CKX8*, *10*,*11* ↑
	*CKX3*, *8*, *NAC2* ↓	*CKX3*, *8*, *NAC2 *↑	
	*CKX1*, *5* ↑	*CKX1*, *5* ↓	*CKX1*, *3*, *NAC2* ↓
yield-related traits	yield ↑	yield ↓	yield ↓
	CKX act. spike ↑	CKX act. spike ↓	CKX act. spike ↓
	root =	root =	root ↓↓
	CKX act. root ↑	CKX act. root ↓	CKX act. root =

High (↑), very high (↑↑), the same (=), slightly low (=↓), the same, lower or higher (=↓↑), low (↓) or very low (↓↓) expression levels, CKX activity or yield-related traits. Relative expression levels: high—1.5–2 higher than in mother; very high—above twice as high as in mother; low—below 0.4 than in mother.

**Table 2:** Positive (+) or negative (−) correlations between *TaCKX* GFMs and *NAC2* expression in spikes, in spikes and roots, and yield-related traits of mother (M) + F_2_ and pater (P) + F_2_ from different crosses.

	CKX Spike		CKX Spike/Root		CKX Spike/Yield-Related Traits	
	M + F_2_	P + F_2_	M + F_2_	P + F_2_	M + F_2_	P + F_2_
C1 M1 + P1	+*CKX1*, 5 *		−*CKX1*/*11*			
	+*CKX2.1*	+*CKX2.1*			+TGW	+SN
	+*CKX2.2.2*	+*CKX2.2.2* +*CKX2.2.2*			+SN	+SN +TGW
	+*CKX5*, *11*	+*CKX5*, *11*! +*CKX5*, *9*		−*CKX5*/1 * +*CKX5*/5 *	−GN, + RM	+SES!
		+*CKX9*, *11*		−*CKX9*/1 *		+SES
	+*CKX10, 11*, *+CKX10*, *11*, *NAC2*! *	+*CKX10*, *11*		+*CKX10*/*8* −*CKX10*/*NAC2*	+TGW	
	+*CKX11*, NAC2 *		+*CKX11/8*! −*CKX11/11*	+*CKX11/8*! −*CKX11/*1	−SN, −GY, −RW	+SES
				−NAC2/3 * −*NAC2/NAC2*!	GY,-SL, +TGW, +RM!	
C2 M1 + P2	+*CKX1*, *11*	+*CKX1*, *2.1*, *2.2.2*, NAC2 *	+*CKX1/5*, 10 *		+PH	
	+*CKX2.1*, 10 *	+*CKX2.1*, *2.2.2*!, NAC2 *				
	+*CKX2.2.2*, 9 *	+*CKX2.2.2*, 10 *			+GN!	
	+*CKX5*	+*CKX5*, *10*			+SES	
	+*CKX9*, *11*		−*CKX9/8*!, −*CKX9/11*!	−*CKX9/8*!, −*CKX9/11*!		+SN, +SES
			−*CKX10/8*! −*CKX10/11*! −*CKX10/NAC2*	−*CKX10/8* −*CKX10*, *11*!		
	*CKX11*				+PH	
	*NAC2*				+ES, −SL, +RM	
C3 M2 + P3	+*CKX1*, *NAC2*	+*CKX1*, *NAC2* +*CKX1*, *11*	+CKX1/1 *			
	+*CKX2.1*, *NAC2*!	−*CKX2.1*, *9* *		+*CKX2.1/11*! *		
	−*CKX5*, *9*				−GY	
			+*CKX9/10*	−*CKX9/8* −*CKX9/11*!		
	+*CKX10*, *11*		+*CKX10/8*, *11*!		−SL	
			−CKX11/3 * +*CKX11/11*		+SN	
				−*NAC2/3*! *		−SN, −SES!
C4 M2 + P4	+*CKX1*	+*CKX1*,*11*			+ES	
	+*CKX2.2.2*		+*CKX2.2.2/10*!	+*CKX2.2.2/10*	+SN	
			−CKX9/1 *		+SES, +SL	
			−*CKX11/1*!, 3 * +*CKX11/8*	+CKX11/NAC2 *	+PH!	
			−*NAC2/NAC2*			
C5 M2 + P2	+*CKX2.1*, *NAC2*	+*CKX2.1*, *NAC2* +*CKX2.1*, *2.2.2* * −*CKX2.1*, *9* *			+SN, +ES	
	+*CKX2.2.2*, *11* *					
	+*CKX5*, *9*! +*CKX5*, *10*	+*CKX5*, *10*!				
				+*CKX11/11*		
			−*NAC2/NAC2*			

*5*, *9*, *11*…—*TaCKX* genes; +/−—positive or negative correlation coefficients ≥ 0.65; !—correlation coefficient ≥ 0.80; *—group-specific correlations; GN—grain number, PH—plant height, ES—empty spikes, SN—spike number, TGW—thousand grain weight, SES—semi-empty spikes, RM—root mass, GY—grain yield, SL—spike length; highlighted in blue—specific to mother and F_2_; highlighted in green—specific to pater and F_2_; highlighted in gray—occurring in both groups.

**Table 3:** *TaCKX* GFM and *NAC2* expression in spikes and roots, and yield-related traits of mother (M), pater (P), and their F_2_ progeny from crosses of awned × awnless parent lines (C6) and awnless × awned parent lines (C7). Character colors indicate similar expression patterns and yield-related traits in pater and F_2_ progeny (red) or in mother and F_2_ progeny (green).

	**C6 = P9 × S8 (awned × awnless)**		
	**M**	**P**	**F_2_**
*CKX* expression 7 DAP	*CKX5* ↑↑↑ *CKX10* ↑↑ *CKX1*, *11* ↑	*CKX5* ↓↓↓ *CKX10* ↓↓ *CKX1*, 11 ↓	*CKX5* ↓↓↓
*CKX* expression root	*CKX1*, *8* ↓↓↓	*CKX1* , 8 ↑↑↑	*CKX1 * ↑↑, *8* ↑↑↑
	*CKX5*, *NAC2* ↑↑↑	*CKX5* , *NAC2* ↓↓↓	*CKX5*, *NAC2* ↓↓↓
yield-related traits	yield ↓↓↓	yield ↑↑↑	yield ↑↑↑
	CKX act. spike =	CKX act. spike =	CKX act. spike =
	root =	root =	root =
	**C7 = S8 × P9 (awnless × awned)**		
	**M**	**P**	**F_2_**
*CKX* expression 7 DAP	*CKX5* ↓↓↓ *CKX10* ↓↓↓ *CKX1*, *11* ↓	*CKX5 *↑↑↑ *CKX10 *↑↑↑ *CKX1*, *11 *↑	*CKX5 *↑ *CKX10 *↑↑↑ *CKX1*, *11 *↑
*CKX* expression root	*CKX5*, *NAC2* ↓↓↓	*CKX5*, *NAC2* ↑↑↑	
	*CKX1*, *8* ↑↑↑	*CKX1*, *8 *↓↓↓	*CKX1*, *5*, *8 *↓
yield-related traits	Yield ↑↑↑	Yield ↓↓↓	Yield =↓
	CKX act. spike =	CKX act. spike =	CKX act. spike =↓↑
	root ↑↑	root ↓↓	root =↓↑

High (↑), very high (↑↑), extremally high (↑↑↑), the same (=), slightly low (=↓), the same, lower or higher (=↓↑), low (↓), very low (↓↓), or extremally low (↓↓↓) expression levels, CKX activity or yield-related traits. Relative expression levels: high—1.5–2 higher than in mother; very high—above twice as high as in mother; extremally high—several times higher as in mother; low—below 0.4 than in mother.

**Table 4:** Positive (+) or negative (−) correlations between the expression of *TaCKX* GFM and *NAC2*, as well as yield-related traits, in the groups of M + F_2_ and P + F_2_ of reciprocal crosses of parents: awned × awnless (C6) and awnless × awned (C7). The same correlations between genes in M + F_2_ and P + F_2_ are in bold. The opposing correlations are colored red.

	C6 M Awned + F_2_	C6 P Awnless + F_2_	C7 M Awnless + F_2_	C7 P Awned + F_2_
*CKXs* spike × *CKXs* spike	+ *CKXs* × *CKXs*	+ *CKXs* × *CKXs*	+ *CKXs* × *CKXs*	+ *CKXs* × *CKXs*
*CKXs* spike × *CKXs* root	+ *CKX1* × *CKX5*, *NAC2* + *CKX5* × *CKX5 × NAC2* + *CKX9* × *CKX5*, *NAC2* + *CKX10* × *CKX5* + *CKX11* × *CKX5*, *NAC2* + *NAC2* × *CKX5*, *NAC2* **− C*KX1* × *CKX10*** − C*KX2.1 × CKX1* − C*KX5* × *CKX1*, *8*, *10*, *11* − C*KX 9* × *CKX1*, *8* *− CKX 10 x CKX8* *− CKX 11 × CKX1*, *8*, *10* − *NAC2* × *CKX1*, *8*	**− C*KX1* × *CKX10***	**+ *CKX2.1 × CKX5*** **+ *CKX2.1 × CKX8*** + *CKX2.1 × CKX1*, *3* **− C*KX2.2.2 × CKX11***+ *CKX5* × *CKX1*, *3* **+ *CKX5* × *CKX5*** **+ *CKX5* × *CKX8*** + *CKX9* × *CKX1*, *3* **+ *CKX9* × *CKX5*** **+ *CKX9* × *CKX8*** + *CKX10* × *CKX3* **+ *CKX10* × *CKX5*** **+ *CKX10* × *CKX8*** **+ *CKX11* × *CKX5*** **+ *CKX11* × *CKX8*** *+ NAC2 × CKX1*, *3* ***+ NAC2 × CKX5*** ***+ NAC2 × CKX8***	− *CKX1 × CKX1*, *3* **+ *CKX2.1 × CKX5*** **− C*KX2.1 × CKX8*** − C*KX2.1 × CKX10*, *11* + *CKX2.1 × NAC2* − C*KX2.2.2 × CKX8* **− C*KX2.2.2 × CKX11*** **+ *CKX5* × *CKX5*** **− C*KX5* × *CKX8*** − C*KX5* × *CKX10*, *11* + *CKX5* × *NAC2* + *CKX9* × *NAC2* **+ *CKX9* × *CKX5*** **− C*KX9* × *CKX8*** − C*KX9* × *CKX11* **+ *CKX10* × *CKX5*** **− C*KX10* × *CKX8*** **+ *CKX11* × *CKX5*** **− C*KX11* × *CKX8*** − C*KX11* × *CKX10*, *11* + *CKX11* × *NAC2* −* NAC2 × CKX1*, *3* ***+ NAC2 × CKX5*** ***+ NAC2 × CKX8*** − *NAC2* × *CKX10*, *11* *+ NAC2 × NAC2*
*CKXs* root × *CKXs* root	+ *CKX1* × *CKX11* + *CKX5* × *NAC2* + *CKX8* × *CKX10* − C*KX1 × CKX5* − C*KX5* × *CKX8*, *10* − C*KX8* × *NAC2* *− CKX10 × NAC2*	+ *CKX1 × CKX5*	+ *CKX1* × *CKX3*, *5* + *CKX5* × *CKX8* + *CKX3* × *CKX5*, *NAC2*	+ *CKX5* × *NAC2* **− C*KX5* × *CKX8*** − C*KX5* × *CKX10*, *11* + *CKX8 × CKX11* − C*KX8* × *NAC2* *− CKX10* × *NAC2* *− CKX11 × NAC2*

**Table 5:** Positive (+) and negative (−) correlations between yield-related traits and *TaCKX* GFM and *NAC2* expression in spikes and roots in two groups of M + F_2_ and P + F_2_ of reciprocal crosses of awned and awnless parents (C6, C7). Divergent correlations are colored red; same correlations are highlighted by gray; lc—lack of correlation with *TaCKX* and *NAC2* genes.

	C6 M Awned + F_2_	C6 P Awnless + F_2_	C7 M Awnless + F_2_	C7 P Awned + F_2_
yield-related traits × *CKXs* spike	− plant height × *CKX5*, *9*, *10*, *11*, *NAC2* − spike number × *CKX5*, *9*, *10*, *11*, *NAC2* semi-empty spike × lc − grain number × *CKX5*, *9*, *10*, *11*, *NAC2* spike length × lc − grain yield × *CKX5*, *9*, *10*, *11*, *NAC* − TGW × *CKX5*, *9*, *10*, *11*, *NAC2* − root weight × *CKX1*, *2.1*, *2.2.2* − root weight × *CKX5*, *9*, *10*, *11*, *NAC2*	− semi-empty spike × *CKX2.1*, *2.2.2*, *10*, *NAC2* + spike length × *CKX1*, *9*, *11*, *NAC2* − root weight × *CKX1*, *2.1*, *2.2.2*	+ spike length × *CKX1*, *2.1*, *5*, *9*, *10*, *11*, *NAC2*	− plant height × *CKX1*, *2.1*, *2.2.2*, *5*, *9*, *10*, *11*, *NAC2* − spike number × *CKX2.1*, *2.2.2*, *5*, *9*, *10*, *11*, *NAC2* + semi-empty spike × *CKX1*, *2.1*, *2.2.2*, *9*, *10*, *NAC2* − grain number × *CKX1*, *2.1*, *2.2.2*, *5*, *9*, *10*, *11*, *NAC2* spike length × lc − grain yield × *CKX2.1*, *2.2.2*, *5*, *9*, *10*, *11*, *NAC2* − TGW × *CKX1*, *2.1*, *2.2.2*, *5*, *9*, *10*, *11*, *NAC2* − root weight × *CKX1*, *2.2.2*, *5*, *9*, *10*, *11*, *NAC2*
yield-related traits × *CKXs* root	+ plant height × *CKX1*, *8*, *10* − plant height × *CKX5*, *NAC2* + spike number × *CKX3*, *8* − spike number × *CKX5* semi-empty spike × lc + grain number × *CKX3*, *8*, *10* − grain number × *CKX5* − grain yield × *CKX5* − TGW × *CKX5* + TGW × *CKX8* spike length × lc + root weight × *CKX1*, *8* − root weight × *CKX5*	+ spike number × *NAC2*	+ spike length × *CKX5*, *8*	+ plant height × *CKX8*, *10*, *11* − plant height × *CKX5*, *NAC2* + spike number × *CKX8*, *11* − spike number × *NAC2* + semi-empty spike × *CKX5* − semi-empty spike × *CKX1*, *8*, *10*, *11* + grain number × *CKX8*, *11* − grain number × *lc* + grain yield × *CKX8*,*11* − grain yield × *CKX5*, *NAC2* + TGW × *CKX1*, *8*, *11* − TGW × *CKX5*, *NAC2* + spike length × *CKX11* − spike length × *CKX3* + root weight × *CKX1*, *8*, *10*, *11* − root weight × *CKX5*

## Data Availability

All data generated or analyzed during this study are included in this published article and its Supplementary Materials files.

## Supplementary Materials

The supporting information can be downloaded at https://www.mdpi.com/article/10.3390/ijms25063553/s1.
