# Supplementary material for: High- or Low-Yielding F2 Progeny of Wheat Is Result of Specific TaCKX Gene Coexpression Patterns in Association with Grain Yield in Paternal Parent
Source: Int J Mol Sci. 2024 Mar 21;25(6):3553. doi: 10.3390/ijms25063553 (PMC10970972; doi:10.3390/ijms25063553)
Supplement: Supplementary file 1 [file ijms-25-03553-s001.zip › ijms-2895202-supplementary.pdf]

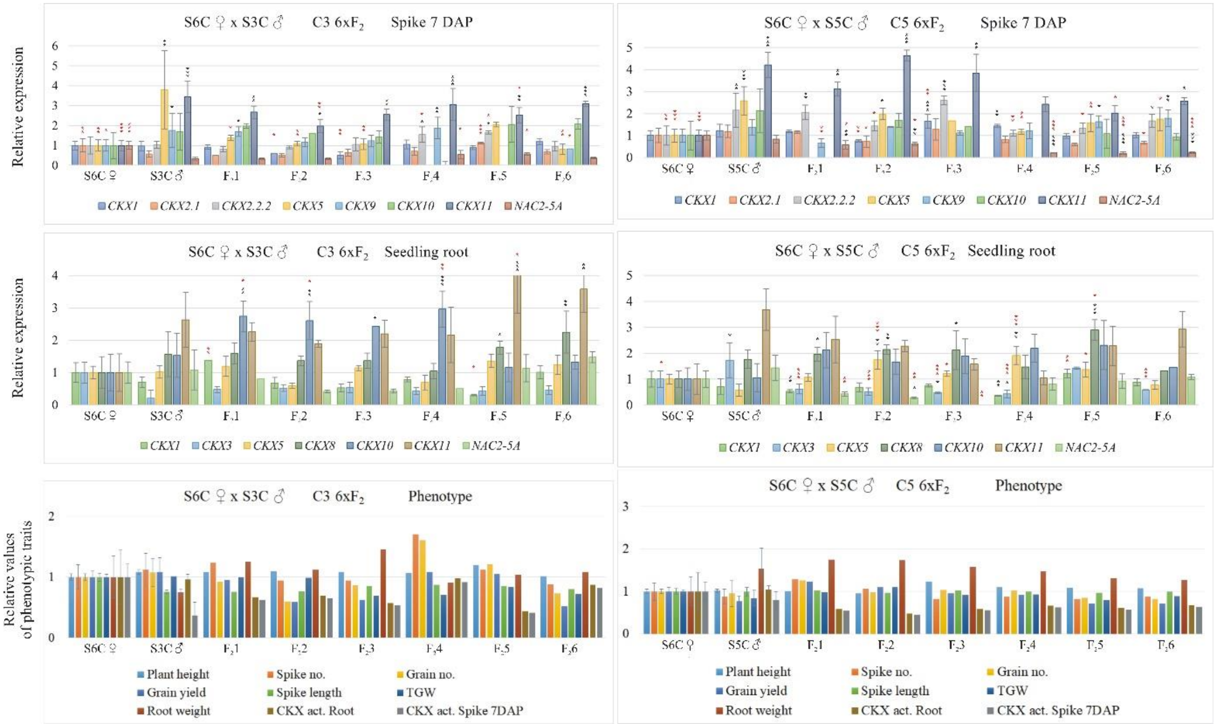

Figure S1. *TaCKX* GFM and *NAC2* expression patterns in 7 DAP spikes, seedling roots, and phenotypic traits in mother, pater, and their six F_2 progeny, from cresses of S6C x S3C (C3), S6C x S12B (C4*) and S6C x S5C (C5). * The data for the C4 cross have already been presented in Szala et al. [24], where S16C was a component of the reciprocal cross.

Table S1. Measurement data of phenotypic traits presented in Figure 1, 2 and S1, in which data were related to mother set as 1.00.

		Plant height [cm]		Spike no.		Semi-empty spikes		Grain no.		Grain yield [g]		Spike length [cm]		TGW		Root weight [mg]		CKX act. Spike 7DAP		CKX act. Root	
		Average for parents	OD for parents	Average for parents	OD for parents	Average for parents	OD for parents	Average for parents	OD for parents	Average for parents	OD for parents	Average for parents	OD for parents	Average for parents	OD for parents	Average for parents	OD for parents	Average for parents	OD for parents	Average for parents	OD for parents
C1	S12B ♀	75.0	5.0	7.3	0.6	0.018	0.016	248.3	41.0	11.8	1.0	10.0	0.2	47.9	4.3	40.7	18.3	0.575	0.052	1.062	0.592
	S6C ♂	71.0	3.6	5.7	1.2	0.000	0.000	173.0	9.2	8.8	0.8	9.6	0.6	50.5	2.2	48.7	16.9	1.057	0.214	1.123	0.440
	$F_2$1	75.0		6.0		0.014		202.0		10.3		10.7		50.8		56.0		0.709		0.979	
	$F_2$2	70.0		6.0		0.027		182.0		8.5		10.2		46.8		66.0		0.961		0.746	
	$F_2$3	78.0		6.0		0.027		129.0		6.3		9.5		48.6		59.0		1.292		1.145	
	$F_2$4	52.0		1.0		0.014		2.0		0.1		4.0		50.0		67.0		0.197		0.681	
	$F_2$5	77.0		7.0		0.000		283.0		13.8		10.6		48.8		15.0		0.962		0.594	
	$F_2$6	76.0		5.0		0.000		199.0		9.0		12.9		45.0		10.0		0.555		0.784	
C2	S12B ♀	75.0	5.0	7.3	0.6	0.018	0.016	248.3	41.0	11.8	1.0	10.0	0.2	47.9	4.3	40.7	18.3	0.575	0.052	1.062	0.592
	S5C ♂	72.3	2.5	5.0	1.0	0.005	0.008	165.0	52.6	6.7	1.1	9.5	1.0	42.7	9.1	74.7	25.0	1.109	0.203	0.891	0.084
	$F_2$1	77.0		6.0		0.027		225.0		10.0		11.2		44.4		18.0		0.835		0.693	
	$F_2$2	81.0		7.0		0.000		223.0		11.1		9.8		49.6		79.0		1.234		1.098	
	$F_2$3	78.0		7.0		0.027		202.0		9.2		8.6		45.7		83.0		0.609			
	$F_2$4	86.0		6.0		0.000		203.0		10.0		9.6		49.1		90.0		1.157		0.800	
	$F_2$5	81.0		3.0		0.014		25.0		0.9		7.5		37.6		90.0		1.259		1.772	
	$F_2$6	71.0		4.0		0.000		102.0		5.0		8.8		49.2		106.0		0.460		1.482	
C3	S6C ♀	71.0	3.6	5.7	1.2	0.000	0.000	173.0	9.2	8.8	0.8	9.6	0.6	50.5	2.2	48.7	16.9	1.057	0.214	1.123	0.440
	S3C ♂	77.0	2.6	6.3	1.5	0.014	0.014	185.7	39.9	9.5	2.0	7.3	0.3	51.1	0.1	36.3	2.5	1.013	0.221	0.416	0.006
	$F_2$1	68.0		7.0		0.014		74.0		3.4		5.0		45.4		113.0		0.975		0.707	
	$F_2$2	76.0		7.0		0.000		233.0		11.7		8.5		50.3		64.0		1.328		0.734	
	$F_2$3	77.0		12.0		0.069		369.0		9.7		8.8		26.3		35.0		1.076		0.604	
	$F_2$4	77.0		8.0		0.000		239.0		9.5		7.7		39.8		54.0		1.531		1.034	
	$F_2$5	66.0		3.0		0.000		39.0		1.5		6.0		39.5		78.0		2.057		0.470	
	$F_2$6	86.0		8.0		0.000		271.0		13.0		8.0		47.9		34.0		2.319		0.925	

C4	S6C ♀	71.0	3.6	5.7	1.2	0.000	0.000	173.0	9.2	8.8	0.8	9.6	0.6	50.5	2.2	48.7	16.9	1.057	0.214	1.123	0.440
	S12B ♂	75.0	5.0	7.3	0.6	0.018	0.016	248.3	41.0	11.8	1.0	10.0	0.2	47.9	4.3	40.7	18.3	0.575	0.052	1.062	0.592
	$F_2$1	73.0		9.0		0.000		257.0		12.9		9.4		50.0		69.0		0.792		1.039	
	$F_2$2	72.0		8.0		0.041		352.0		17.9		11.6		50.7		71.0		0.601		1.049	
	$F_2$3	69.0		8.0		0.000		235.0		12.1		9.6		51.3		41.0		0.878		1.084	
	$F_2$4	76.0		4.0		0.000		147.0		8.0		11.0		54.1		96.0		0.476		1.234	
	$F_2$5	78.0		6.0		0.000		250.0		11.5		12.2		46.0		47.0		1.173		0.568	
	$F_2$6	75.0		4.0		0.055		120.0		6.9		11.3		57.2		67.0		1.317		1.988	
C5	S6C ♀	71.0	3.6	5.7	1.2	0.000	0.000	173.0	9.2	8.8	0.8	9.6	0.6	50.5	2.2	48.7	16.9	1.057	0.214	1.123	0.440
	S5C ♂	72.3	2.5	5.0	1.0	0.005	0.008	165.0	52.6	6.7	1.1	9.5	1.0	42.7	9.1	74.7	25.0	1.109	0.203	0.891	0.084
	$F_2$1	67.0		13.0		0.055		374.0		17.9		8.8		47.9		99.0		1.157		0.616	
	$F_2$2	68.0		9.0		0.027		239.0		13.0		9.3		54.5		87.0		1.286		0.506	
	$F_2$3	72.0		6.0		0.000		219.0		13.2		10.0		60.5		67.0		1.230		0.624	
	$F_2$4	64.0		3.0		0.014		50.0		2.6		8.8		52.8		100.0		1.806		0.704	
	$F_2$5	71.0		2.0		0.014		11.0		0.4		8.0		38.2		94.0		0.819		0.646	
	$F_2$6	81.0		6.0		0.000		266.0		12.8		11.0		47.9		43.0		0.823		0.719	
C6	P9 ♀	40.0	1.0	4.7	2.1	1.667	1.528	104.3	72.7	2.2	1.0	7.8	1.0	24.5	12.5	92.7	12.1	1.009	0.180		
	S8 ♂	77.0	2.6	6.3	3.1	0.000	0.000	278.7	153.4	10.8	5.6	12.1	1.2	40.0	4.2	76.3	7.6	0.985	0.101		
	$F_2$1	80.0		8.0		2.000		273.0		12.8		10.9		47.0		101.0		0.946			
	$F_2$2	56.0		4.0		2.000		90.0		2.4		6.7		26.6		94.0		0.871			
	$F_2$3	55.0		6.0		1.000		86.0		3.1		6.8		35.8		114.0		0.970			
	$F_2$4	67.0		6.0		0.000		215.0		7.6		10.5		35.1		87.0		0.857			
	$F_2$5	83.0		7.0		0.000		309.0		12.7		9.7		41.1		64.0		1.247			
	$F_2$6	74.0		9.0		0.000		313.0		9.8		9.8		31.3		74.0		0.780			
C7	S8 ♀	77.0	2.6	6.3	3.1	0.000	0.000	278.7	153.4	10.8	5.6	12.1	1.2	40.0	4.2	76.3	7.6	0.985	0.101		
	P9 ♂	40.0	1.0	4.7	2.1	1.667	1.528	104.3	72.7	2.2	1.0	7.8	1.0	24.5	12.5	92.7	12.1	1.009	0.180		
	$F_2$1	75.0		7.0		0.000		247.0		11.5		8.8		46.5		86.0		0.935			
	$F_2$2	74.0		6.0		0.000		220.0		10.3		9.8		46.6		85.0		1.402			
	$F_2$3	62.0		3.0		0.000		63.0		1.7		7.5		27.3		90.0		0.820			
	$F_2$4	72.0		7.0		0.000		222.0		8.6		8.2		38.9		66.0		1.219			
	$F_2$5	79.0		7.0		0.000		247.0		12.3		8.1		49.8		70.0		0.844			
	$F_2$6	54.0		3.0				58.0		2.9		7.5		49.3		82.0		0.584			

Table S2. Correlation coefficients between *TaCKX* GFM and *NAC2* expression in spikes and roots, CKX activity and yield-related traits of M + F2 and P + F2 from different crosses.

C1 & C2 & C3 & C4 & C5		*CKX2.1* Spike	*CKX2.2.2* Spike	*CKX5* Spike	*CKX9* Spike	*CKX10* Spike	*CKX11* Spike	*NAC2-5A* Spike	*CKX1* Root	*CKX3* Root	*CKX5* Root	*CKX8* Root	*CKX10* Root	*CKX11* Root	*NAC2-5A* Root	Aktywność bezwzględna Korzeń	Aktywność bezwzględna . Kłos 7DAP	Plant height	Spike number	Empty spike number	Semi-empty spike number	Grain number	Grain yield	Spike length	TGW
CKX1 Spike M1 + P1	M + F_2			0.73			0.63							-0.67			0.73					-0.61			
	P + F_2			0.39			-0.12							-0.43			0.58					-0.25			
CKX1 Spike M1 + P2	M + F_2	0.24	0.13				0.67	0.32			0.69		0.75		0.35		0.90	0.67							
	P + F_2	0.74	0.75				0.36	0.67			0.14		0.11		0.63		0.80	0.03							
CKX1 Spike M2 + P3	M + F_2						-0.06	0.68	0.78																
	P + F_2						0.67	0.68	0.52																
CKX1 Spike M2 + P4	M + F_2						0.16							-0.20			0.67			0.71					
	P + F_2						0.68							-0.60			0.87			0.55					
CKX1 Spike M2 + P2	M + F_2		0.51																						
	P + F_2		0.62																						
CKX2.1 Spike M1 + P1	M + F_2		0.05														0.62		0.50						0.65
	P + F_2		0.60														0.33		0.67						0.33
CKX2.1 Spike M1 + P2	M + F_2		-0.26			0.70		0.35							-0.62										
	P + F_2		0.81			0.57		0.71							0.29										
CKX2.1 Spike M2 + P3	M + F_2				-0.52			0.81					-0.67	0.29			-0.03								
	P + F_2				-0.70			0.52					-0.44	0.83			0.69								
CKX2.11 Spike M2 + P4	M + F_2																								
	P + F_2																								
CKX2.1 Spike M2 + P2	M + F_2		0.10		-0.65			0.74											0.65	0.73					
	P + F_2		0.75		-0.75			0.67											0.26	0.41					
CKX2.2.2 Spike M1 + P1	M + F_2																0.37		0.65						-0.05
	P + F_2																0.61		0.74						0.69
CKX2.2.2 Spike M1 + P2	M + F_2				0.72	0.42	0.62					-0.60		-0.60		-0.61			0.61			0.81			
	P + F_2				0.01	0.68	0.22					-0.29		-0.50	0.60	-0.55			-0.02			0.13			
CKX2.2.2 Spike M2 + P3	M + F_2																			-0.10					
	P + F_2																			-0.62					
CKX2.2.2 Spike M2 + P4	M + F_2										0.60	0.64	0.87						0.73						
	P + F_2										0.63	0.56	0.74						0.14						
CKX2.2.2 Spike M2 + P2	M + F_2						0.69																		
	P + F_2						-0.28																		
CKX5 Spike M1 + P1	M + F_2				0.50		0.68		-0.61	-0.33	0.29	0.22		-0.64	-0.37						0.62	-0.69			
	P + F_2				0.76		0.80		-0.78	-0.61	0.66	0.63		0.01	-0.61						0.90	-0.32			
CKX5 Spike M1 + P2	M + F_2					0.43									-0.62						0.78				
	P + F_2					0.67									-0.26						0.31				
CKX5 Spike M2 + P3	M + F_2				-0.68	0.48										-0.68							-0.67		
	P + F_2				0.03	0.62										-0.67							0.02		
CKX5 Spike M2 + P4	M + F_2											-0.02													
	P + F_2											-0.60													
CKX5 Spike M2 + P2	M + F_2				0.84	0.78																			
	P + F_2				0.50	0.87																			
CKX9 Spike M1 + P1	M + F_2						0.38		-0.08												0.29				
	P + F_2						0.72		-0.66												0.66				
CKX9 Spike M1 + P2	M + F_2						0.68					-0.86		-0.82		-0.68		0.60	0.38						
	P + F_2						0.28					-0.95		-0.82		-0.43		0.55	0.66		0.65				
CKX9 Spike M2 + P3	M + F_2										-0.54	-0.35	0.70	-0.49	-0.49		-0.51								
	P + F_2										-0.61	-0.70	0.47	-0.85	-0.60		-0.61								
CKX9 Spike M2 + P4	M + F_2						0.60		-0.72												0.71			0.71	
	P + F_2						0.49		-0.21												0.50			0.42	
CKX9 Spike M2 + P2	M + F_2																								
	P + F_2																								
CKX10 Spike M1 + P1	M + F_2						0.75	0.83				0.61			-0.18										0.73
	P + F_2						0.75	0.59				0.69			-0.69										0.13
CKX10 Spike M1 + P2	M + F_2										-0.33	-0.93	-0.29	-0.93	-0.69						0.64				
	P + F_2										-0.62	-0.74	-0.62	-0.90	-0.36						0.31				
CKX10 Spike M2 + P3	M + F_2						0.75			-0.69		0.91		0.88		-0.76								-0.78	
	P + F_2						0.57			-0.69		-0.13		0.15		0.04								-0.28	
CKX10 Spike M2 + P4	M + F_2																								
	P + F_2																								
CK10 Spike M2 + P2	M + F_2														-0.60	-0.63									
	P + F_2														-0.02	-0.27									
CKX11 Spike M1 + P1	M + F_2							0.79	-0.26			0.88		-0.71					-0.71		0.02		-0.65		
	P + F_2							0.28	-0.69			0.86		0.07					-0.02		0.76		-0.15		
CKX11 Spike M1 + P2	M + F_2																0.66	0.79							
	P + F_2																0.24	-0.08							
CKX11 Spike M2 + P3	M + F_2									-0.67		0.62	0.61	0.70					0.67					-0.63	
	P + F_2									-0.64		-0.37	-0.41	-0.14					-0.13					0.01	
CKX11 Spike M2 + P4	M + F_2								-0.83	-0.69		0.73	0.62		-0.29		0.04	0.83						0.64	
	P + F_2								-0.51	-0.33		0.62	0.35		0.67		0.60	0.25						0.49	
CKX11 Spike M2 + P2	M + F_2									-0.62				0.51		-0.74									
	P + F_2									-0.02				0.75		0.22									
NAC2-5A Spike M1 + P1	M + F_2									-0.32		0.66			-0.65								-0.66	-0.71	0.70
	P + F_2									-0.78		0.61			-0.88								-0.39	-0.55	0.50
NAC2-5A Spike M1 + P2	M + F_2															0.78				0.73		-0.64		-0.77	
	P + F_2															0.36				0.21		-0.46		-0.35	
NAC2-5A Spike M2 + P3	M + F_2									-0.48									-0.31		-0.49	-0.34			
	P + F_2									-0.90									-0.75		-0.84	-0.62			
NAC2-5A Spike M2 + P4	M + F_2														-0.68										
	P + F_2														-0.63										
NAC2-5A Spike M2 + P2	M + F_2												-0.35		-0.71										
	P + F_2												-0.66		0.46										

Highlighted in blue – specific to mother and F2; highlighted in green – specific to pater and F2; highlighted in yellow – occurring in both groups.

Table S3. Correlation coefficients between the expression of *TaCKX* GFM and *NAC2* in spikes or spikes per roots, as well as yield-related traits in the groups of M + F_2 and P + F_2 of reciprocal crosses of awned x awnless (C6) and awnless x awned parents (C7).

**7K (C6 awned x awnless)**		*CKX2.1* Spike	*CKX2.2.2* Spike	*CKX5* Spike	*CKX9* Spike	*CKX10* Spike	*CKX11* Spike	*NAC2-5A* Spike	*CKX1* Root	*CKX3* Root	*CKX5* Root	*CKX8* Root	*CKX10* Root	*CKX11* Root	*NAC2-5A* Root	Plant height	Spike number	Empty spike number	Semi-empty spike	Grain number	Grain yield	Spike length	TGW	Root wieght
CKX1 Spike	C6 (M + F_2)	**0.78**	**0.82**	0.71	0.67	0.69	**0.86**	0.67			**0.79**	-0.71	**-0.82**		**0.74**					-0.52	-0.42	0.11		**-0.70**
	C6 (P + F_2)	**0.72**	**0.83**	0.71	0.69	**0.76**	**0.86**	0.69			0.00	-0.18	**-0.79**		0.02					0.60	0.60	0.65		-0.60
CKX2.1 Spike	C6 (M + F_2)		**0.88**	**0.81**	**0.72**	**0.81**		**0.81**	**-0.67**										-0.27				-0.62	**-0.90**
	C6 (P + F_2)		**0.80**	**0.76**	**0.77**	**0.90**		0.81	-0.13										**-0.79**				0.24	**-0.66**
CKX2.2.2 Spike	C6 (M + F_2)			0.64	**0.76**	**0.83**	0.62	**0.71**			0.61	-0.68							-0.39					**-0.97**
	C6 (P + F_2)			0.75	**0.74**	**0.83**	0.62	0.69			-0.25	-0.11							**-0.78**					**-0.88**
CKX5 Spike	C6 (M + F_2)				**0.84**	0.75	**0.99**	**0.79**	**-0.93**		**0.98**	**-0.87**	**-0.84**	**-0.82**	**0.90**	**-0.83**	**-0.80**			-0.70	-0.68		**-0.84**	-0.69
	C6 (P + F_2)				**0.86**	**0.82**	0.64	**0.96**	0.11		-0.61	0.21	-0.43	-0.36	-0.57	0.07	-0.35			0.12	0.00		0.17	-0.31
CKX9 Spike	C6 (M + F_2)					**0.99**	**0.90**	**0.92**	**-0.76**		**0.92**	**-0.84**	-0.70		**0.73**	**-0.88**	**-0.82**			**-0.72**	**-0.70**	-0.41	**-0.89**	**-0.73**
	C6 (P + F_2)					0.60	**0.95**	**0.92**	0.39		-0.08	-0.27	-0.59		-0.31	0.37	-0.01			0.33	0.33	**0.72**	0.26	-0.36
CKX10 Spike	C6 (M + F_2)						**0.84**	**0.86**	-0.71		**0.84**	**-0.82**	-0.61		0.62	**-0.82**	**-0.79**		-0.46	-0.67	-0.64		**-0.86**	**-0.72**
	C6 (P + F_2)						0.42	**0.84**	0.28		-0.14	-0.15	-0.54		-0.25	0.35	0.54		**-0.87**	0.64	0.58		0.06	-0.24
CKX11 Spike	C6 (M + F_2)							**0.86**	**-0.79**		**0.97**	**-0.93**	**-0.76**		**0.88**	**-0.86**	**-0.86**			**-0.73**	-0.69	0.06	**-0.89**	**-0.77**
	C6 (P + F_2)							**0.77**	0.15		-0.19	-0.12	-0.49		-0.24	0.35	0.15			0.42	0.34	**0.73**	0.09	-0.35
NAC2-5A Spike	C6 (M + F_2)								**-0.80**		**0.96**	**-0.92**	-0.75		**0.90**	**-0.88**	**-0.86**		-0.31	**-0.72**	-0.69	-0.25	**-0.88**	**-0.80**
	C6 (P + F_2)								0.50		-0.07	-0.15	-0.58		-0.25	0.43	0.24		-0.70	0.50	0.44	0.67	0.20	-0.45

**8K (C7 awnless x awned)**		*CKX2.1* Spike	*CKX2.2.2* Spike	*CKX5* Spike	*CKX9* Spike	*CKX10* Spike	*CKX11* Spike	*NAC2-5A* Spike	*CKX1* Root	*CKX3* Root	*CKX5* Root	*CKX8* Root	*CKX10* Root	*CKX11* Root	*NAC2-5A* Root	Plant height	Spike number	Empty spike number	Semi-empty spike number	Grain number	Grain yield	Spike length	TGW	Root wieght
CKX1 Spike	C7 (M + F_2)	**0.79**		0.75	**0.87**	**0.71**	**0.77**	**0.93**	0.27	0.12		0.67				0.41			0.17	0.43		**0.92**	-0.45	-0.19
	C7 (P + F_2)	**0.81**		0.75	**0.85**	0.64	**0.77**	**0.92**	**-0.73**	**-0.67**		-0.45				**-0.67**			**0.79**	-0.60		-0.14	**-0.78**	**-0.64**
CKX2.1 Spike	C7 (M + F_2)		**0.81**	**0.95**	**0.99**	0.68	**0.90**	**0.97**	**0.83**	**0.71**	**0.93**	**0.79**	-0.02	-0.55	0.22	0.50	0.20		0.17	0.27	0.35	**0.85**	-0.17	
	C7 (P + F_2)		**0.83**	**0.91**	**0.90**	**0.80**	**0.94**	**0.97**	-0.43	-0.30	**0.98**	**-0.87**	**-0.77**	**-0.80**	**0.98**	**-0.92**	**-0.67**		**0.79**	**-0.78**	**-0.73**	-0.49	**-0.89**	
CKX2.2.2 Spike	C7 (M + F_2)			**0.86**		0.69	0.60		0.38			0.31		**-0.86**		0.30	0.11		0.17	0.16	0.02		-0.28	-0.45
	C7 (P + F_2)			0.75		0.69	0.60		-0.62			**-0.81**		**-0.88**		**-0.85**	-0.62		**0.79**	**-0.70**	**-0.72**		-0.62	**-0.78**
CKX5 Spike	C7 (M + F_2)				**0.92**	0.53	**0.77**	**0.90**	**0.95**	**0.92**	**0.99**	0.79	-0.26	-0.45	0.56	0.66	0.17		bd	0.32	0.40	**0.79**	0.04	-0.38
	C7 (P + F_2)				**0.83**	0.75	**0.99**	**0.79**	-0.63	-0.48	**1.00**	**-0.90**	-0.81	**-0.82**	0.98	**-0.86**	**-0.80**		**0.87**	**-0.89**	-0.66	-0.51	**-0.85**	**-0.79**
CKX9 Spike	C7 (M + F_2)					0.60	**0.95**	**0.93**	**0.75**	**0.82**	**0.88**	**0.80**	0.01	-0.55	0.13	0.45	0.09		bd	0.33	0.35	**0.93**	-0.25	-0.14
	C7 (P + F_2)					**0.99**	**0.91**	**0.93**	-0.62	-0.24	**0.94**	**-0.85**	-0.60	**-0.71**	0.81	**-0.91**	**-0.85**		**0.82**	**-0.78**	**-0.70**	-0.59	**-0.90**	**-0.82**
CKX10 Spike	C7 (M + F_2)						0.42	**0.84**	0.66	**0.80**	**0.82**	**0.78**		-0.51	0.38	0.47	0.40		bd	0.52	0.65	0.60	-0.14	-0.11
	C7 (P + F_2)						**0.84**	**0.86**	-0.57	-0.03	**0.87**	**-0.79**		-0.62	0.69	**-0.86**	**-0.74**		**0.85**	**-0.71**	-0.65	-0.60	**-0.85**	**-0.79**
CKX11 Spike	C7 (M + F_2)							**0.78**	0.55	0.70	**0.73**	**0.77**	0.10	-0.52	0.09	0.37	0.18		bd	0.31	0.34	**0.91**	-0.29	-0.03
	C7 (P + F_2)							**0.87**	-0.63	-0.47	**1.00**	**-0.89**	**-0.73**	**-0.79**	0.95	**-0.90**	**-0.78**		**0.82**	**-0.83**	**-0.71**	-0.57	**-0.90**	**-0.81**
NAC2-5A Spike	C7 (M + F_2)								**0.87**	**0.87**	**0.95**	**0.79**	-0.06	-0.54	0.15	0.48	0.18		bd	0.38	0.44	**0.86**	-0.21	-0.17
	C7 (P + F_2)								-0.62	-0.51	**1.00**	**-0.89**	**-0.74**	**-0.79**	0.96	**-0.92**	**-0.75**		**0.82**	**-0.68**	**-0.70**	-0.47	**-0.88**	**-0.84**

Highlighted in blue – specific to mother and F_2; highlighted in green – specific to pater and F_2; highlighted in yellow – occurring in both groups. Bold – significant at $p \geq 0.01$.
